# Post-Translational Modifications of RelB NF-κB Subunit and Associated Functions

**DOI:** 10.3390/cells5020022

**Published:** 2016-05-04

**Authors:** Véronique Baud, Davi Collares

**Affiliations:** NF-κB, Differentiation and Cancer, Université Paris Descartes, Sorbonne Paris Cité, 75014 Paris, France

**Keywords:** NF-kappaB, RelB, post-translational modifications, cell motility, phosphorylation, ubiquitination, SUMOylation, NF-κB alternative pathway

## Abstract

The family of NF-κB transcription factors plays a key role in diverse biological processes, such as inflammatory and immune responses, cell survival and tumor development. Beyond the classical NF-κB activation pathway, a second NF-κB pathway has more recently been uncovered, the so-called alternative NF-κB activation pathway. It has been shown that this pathway mainly controls the activity of RelB, a member of the NF-κB family. Post-translational modifications, such as phosphorylation, acetylation, methylation, ubiquitination and SUMOylation, have recently emerged as a strategy for the fine-tuned regulation of NF-κB. Our review discusses recent progress in the understanding of RelB regulation by post-translational modifications and the associated functions in normal and pathological conditions.

## 1. Introduction

Nuclear factor κB (NF-κB) was first described in 1986 as nuclear factor binding the kappa light chain enhancer in B cells [1]. Since then, it has been demonstrated to play a central role in the inflammatory and immune responses, but it also controls cell proliferation and protects the cell from apoptosis [2,3,4]. The relevance of NF-κB in tumor maintenance, tumor development and possibly even in tumor initiation is becoming more evident [5,6,7,8] and, recently, activation of NF-κB has been implicated in tumor resistance to chemotherapy and radiotherapy [9]. 

**Figure 1:**
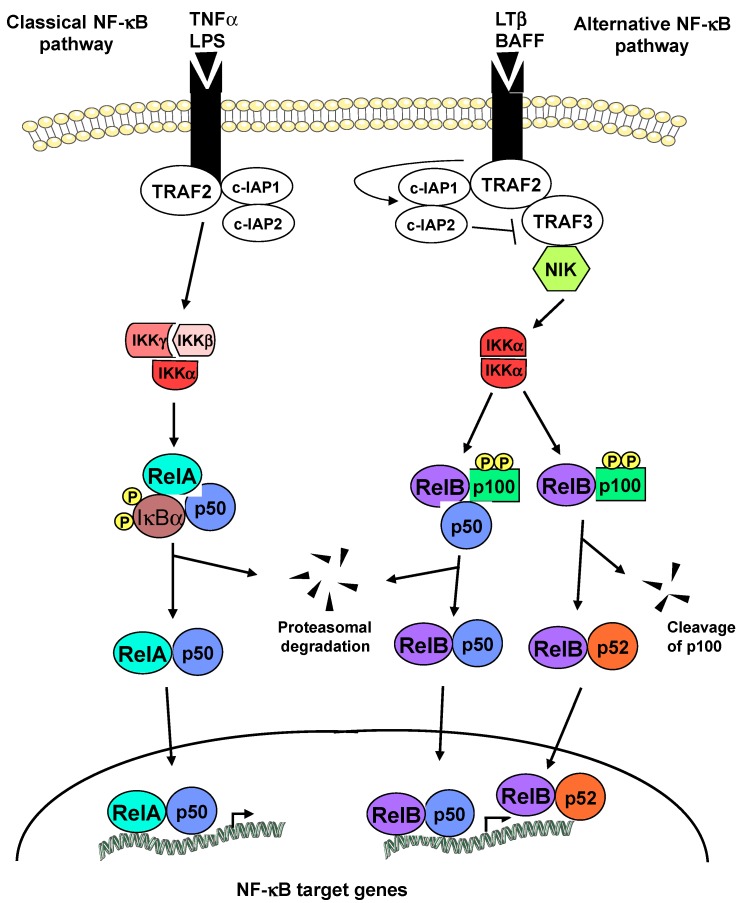
The classical and alternative NF-κB activation pathways. The classical NF-κB pathway (*left*): Activation of various receptors, such as TNFRs, causes phosphorylation of the inhibitory IκB proteins by the IKK complex, leading to their phosphorylation at two specific serine residues, and thereby their degradation by the proteasome 26S. Freed from their inhibitory interaction with the IκBs, RelA- and cRel-containing dimers translocate to the nucleus where they activate the transcription of specific NF-κB target genes. The alternative NF-κB pathway (*right*): Activation of a more restricted set of receptors (e.g., BAFF, lymphotoxin β), causes the degradation of TRAF3 by the cIAP1/2 E3 ligases, leading to the activation of the MAP3K NIK that activates IKKα, subsequently leading to the phosphorylation and proteasome-dependent processing of p100 and ultimately resulting in the release of either RelB/p50 or RelB/p52 dimers.

In mammals, the NF-κB family is composed of five members, RelA (p65), RelB, cRel (Rel), NF-κB1 (p50 and its precursor p105) and NF-κB2 (p52 and its precursor p100) [10]. These proteins form a variety of homo- and hetero-dimers that, in a resting cell, are retained in a latent cytoplasmic form through binding to a member of the inhibitor of NF-κB (IκB) protein family. Upon cell stimulation, NF-κB is activated by two main pathways (Figure 1). The first one is called the classical NF-κB pathway. It involves activation of the IκB kinase (IKK) complex, leading to phosphorylation of IκB proteins and their subsequent ubiquitinylation and degradation by the proteasome [11] (Figure 1, *left*). This releases active complexes to translocate to the nucleus and execute their transcription functions. The classical pathway usually regulates the activity of RelA and cRel containing dimers. It is typically responsible for a strong and rapid NF-κB activating signal in response to stress situations and plays a crucial role in the regulation of inflammation and innate immunity. Inflammatory cytokine tumor necrosis factor α (TNFα), toll-like receptors (TLR), interleukine-1 (IL-1) and lipopolysaccharide (LPS) are some of the stimuli involved in its activation. The second one, the more recently described alternative NF-κB pathway, leads to the activation of RelB-containing dimers (Figure 1, *right*) [7,12,13]. This pathway involves the NF-κB inducing kinase (NIK) that activates IKKα, thereby leading to the phosphorylation and proteasome-dependent processing of p100, resulting in the release of RelB/p50 and RelB/p52 dimers (Figure 1, *right*). It is known to be involved in diverse processes such as lymphoid organogenesis and B cell survival, as well as in the regulation of adaptive immunity. It is activated by a more restricted subset of TNF family members (e.g., lymphotoxin β (LTβ), B-cell activating factor (BAFF) and CD40 ligand).

RelB is the only NF-κB member that cannot homodimerize, and it only triggers potent transcriptional activation when coupled to p50 or p52 [14,15,16,17]. Beyond the alternative NF-κB signaling cascade, RelB-dependent DNA-binding activity is negatively regulated at the nuclear level by several mechanisms, such as trapping in RelA/RelB or p100/RelB complexes [18,19,20], and post-translational modifications (see above). RelB-containing dimers also display DNA-binding specificity [21,22,23]. RelB recruitment to some genes correlates with transcriptional downregulation (IL12-p40), whereas in other cases (EBV-induced molecule 1 ligand chemokine (ELC) and macrophage-derived chemokine (MDC)), it increases transcriptional activity well over the level achieved by RelA or cRel [24]. Altogether, this emphasizes the importance and unique role of RelB.

Analyses of RelB-deficient mice have shown that RelB is essential to the development of medullary epithelium, mature dendritic cell function, and secondary lymphoid tissue organization [25,26,27,28], indicating that RelB exerts a crucial positive effect for these developmental processes that cannot be compensated for by the presence of other NF-κB proteins. RelB-deficient mice also spontaneously develop a generalized persistent non-infectious multi-organ inflammatory syndrome that contributes significantly to their premature mortality [29]. ReB is a critical element involved in dendritic cell maturation and immune tolerance to inflammation [30,31]. ReB also represses expression of immediate-response proinflammatory genes during endotoxin tolerance in monocytes, [32,33,34]. The participation of RelB in non-hematopoietic related function has also emerged. RelB has been shown to play an essential role in limiting the expression of proinflammatory mediators in lipopolysaccharide-induced fibroblasts [35,36], thereby playing an important role in the resolution of acute inflammation. RelB promotes mitochondrial biogenesis in muscle cells [37,38,39], participates in the regulation of the circadian rhythm in murine fibroblasts [40] and supports the xenobiotic-detoxifying pathway in lung fibroblasts [41,42]. RelB also plays an important role in RANKL-induced osteoclastogenesis that cannot be compensated for by RelA [43,44,45]. 

Furthermore, accumulating evidence strongly suggest that an abnormal activity of RelB is involved in the development of both hematopoietic malignancies and solid cancers [13]. Constitutive activation of RelB/p50 dimers participates in the inhibition of DNA-damage-induced apoptosis in certain types of MALT lymphoma [46]. A frequent constitutive RelB DNA-binding activity was reported in a cohort of newly diagnosed multiple myeloma patients [47]. It was demonstrated that RelB plays a crucial role in promoting multiple myeloma cell survival *via* the increased expression of a subset of anti-apoptotic NF-κB target genes (e.g., cIAP2) by a direct transcriptional control [47]. Inhibition of Notch-induced RelB/p52 activity in Hodgkin lymphoma cell lines is associated with apoptosis and decreased expression of cIAP2 [48]. Moreover, bone marrow stem cells (BMSCs) prevent apoptosis of primary B lymphoma cells, at least in part, through RelB-dependent increased expression of NF-κB-dependent anti-apoptotic genes (including cIAP1/2 and XIAP) [49]. Thus, it is likely that the prosurvival effects of RelB observed in multiple myeloma might be generalized to other B-cell neoplasms, especially those addicted to NF-κB. RelB also assisted TEL-JAK2-induced T-cell leukemogenesis [50]. Interestingly, in non-hematopoietic stromal cells, RelB has a role favoring leukemia onset and increasing disease severity.

Abnormal high level of RelB expression has been reported in various solid cancers (e.g., glioblastoma, prostate, breast, bladder and non-small cell lung cancers) and appears to correlate with tumor aggressiveness [51,52,53,54,55]. RelB is the most frequently detected NF-κB subunit in the nucleus of prostate cancer tissue [51]. The level of nuclear RelB correlates with a patient’s Gleason score, suggesting that RelB expression levels are associated with prostate cancer progression. Moreover, RelB exerts a radioprotective role in aggressive prostate cancer cells, at least partially via the induction of the MnSOD gene [56,57]. RelB promotes glioma cell survival and proliferation, and controls invasion independently from RelA [53,58]. In addition, inhibition of RelB in human breast cancer cells reduced cyclin D1 and c-myc expression, slower proliferation, and repressed transformed phenotype [59]. These data suggest that RelB promotes mammary gland carcinogenesis. Higher RelB expression was demonstrated in estrogen receptor α (ERα)-negative breast cancer *versus* ERα-positive one. Moreover, it has been shown that RelB promotes a more invasive phenotype in ERα-negative cancer via induction of the anti-apoptotic BCL2 gene [52]. RelB also favors resistance of these cells to γ-irradiation and the chemotherapeutic agent doxorubicin [60]. RelB mRNA levels were also associated with bladder cancer tumor grade, clinical stage and lymph node metastasis profile [54].

Post-translational modifications are changes or alterations in a protein occurring after the completion of the translational process, either when a functional group is covalently added to the protein, or during the proteolytic and folding processes. These structural changes act as a mechanism for the specification of proteins and increase their variety. Post-translational modifications have emerged as one of the diverse strategies known for to the fine-tuned regulation of NF-κB. Reported modifications targeting NF-κB activity include phosphorylation, acetylation, methylation, ubiquitinylation, SUMOylation, and isomerization of specific amino acid residues, and target either the IKKs, the IκBs, the NF-κB subunits, or critical adaptor proteins that feed into NF-κB [61,62,63,64,65,66,67]. Such modifications influence initiation and duration of NF-κB response, its specificity for a determined signaling cascade, cell-specific response to a certain stimulus and specific gene transcription. Depending on the cell type and stimulus, such modifications activate or repress NF-κB activity [61,62,68]. Among those involving NF-κB transcription factors, site-specific modifications of RelA is by far the most well known [62,63,64,69,70]. Our review discusses recent progress in the understanding on RelB regulation by post-translational modifications (Table 1) and its associated functions.

## 2. Phosphorylation of RelB

### 2.1. Serine 552 and Threonine 84

Marienfeld *et al.* were the first to describe by *in vitro* kinase assays that RelB can be phosphorylated on threonine 84 and serine 552 [71]. Furthermore, TPA-ionomycin-induced RelB phosphorylation was shown to depend on these two specific sites as evaluated by *in vivo* labeling in murine EL-4 T cells. The authors report a marked decrease in RelB protein expression upon TPA-ionomycin stimulation in human peripheral blood T cells and Jurkat cells. In contrast, TNFα has no effect on RelB expression levels. Interestingly, a phosphorylation-defective RelB mutant serine 552 to cysteine and threonine 84 to alanine (S552C/T84A) leads to the stabilization of RelB. Thus, it indicates that TPA-ionomycin-induced S552 and T84 phosphorylation of RelB leads to its degradation. Remarkably, a cleaved form of RelB was best observed upon pretreatment of T-cells by proteasome inhibitors, suggesting that RelB cleavage can precede its degradation by the proteasome. Notably, cleavage of RelB near its N-terminus (after arginine 85) by the paracaspase MALT1 has been reported [76]. However, mutation of serine 552 and threonine 84 did not prevent RelB cleavage by MALT1 in 293T cells, thereby indicating that these two sites do not appear to be involved in MALT1-dependent RelB cleavage [76].

### 2.2. Serine 368

Maier *et al.* identified RelB serine 368 in the C-terminal part of the Rel Homology domain (RHD) as a conserved residue in human and drosophila NF-κB subunits. [72] As evaluated by luciferase reporter assays, both S368A inactivating and S368E phosphomimetic RelB point mutants exhibited a markedly reduced transcriptional activity in RelB-defective murine S107 plasmacytoma cells compared to that seen in wild-type (WT) RelB. It thus suggests that serine 368 alone rather than its phosphorylation is critical for the control of RelB activity. Mutation of serine 368 severely affects RelB dimerization with its interacting partners p50, p52, RelA and p100. Remarkably, absence of serine 368 correlates with a strong decrease in p100 half-life along with an increase in p100 proteolysis into p52. No similar effect was seen with p105. Whether the phosphorylation of serine 368 can occur on endogenous RelB is still unknown. 

### 2.3. Serine 472

**Figure 2:**
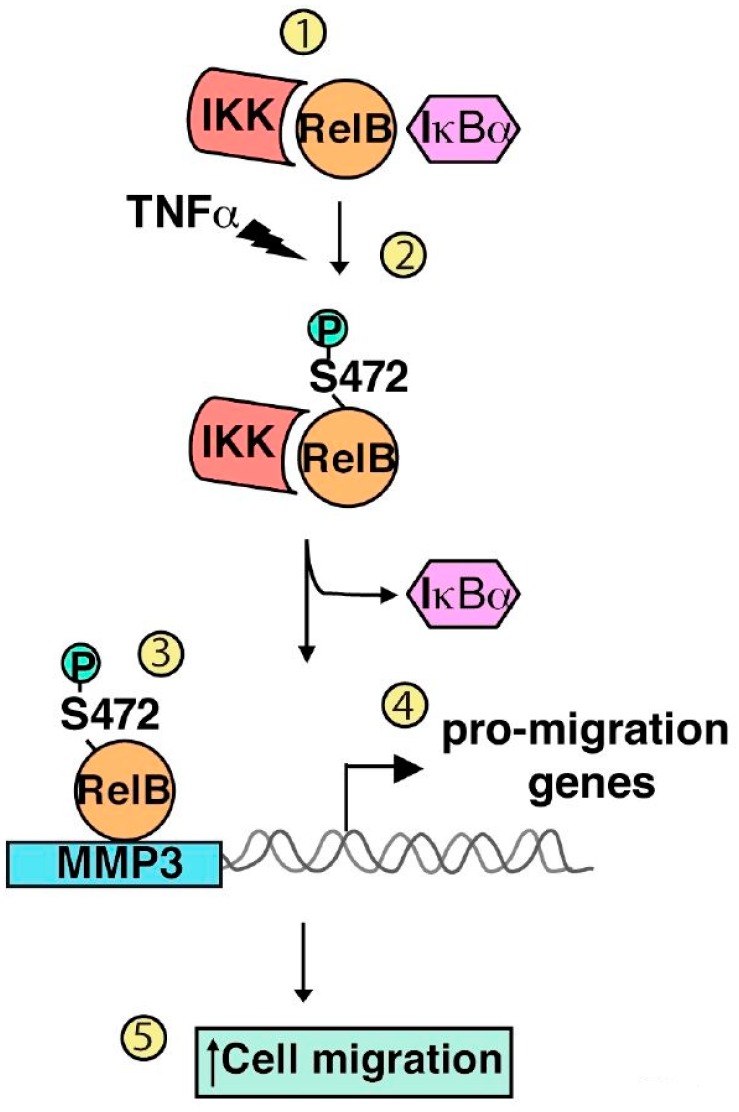
Model for RelB serine-472 phosphorylation acting as an activator of inflammation-mediated cell migration. The IκB kinase (IKK) complex constitutively interacts with the RelB subunit of NF-κB [1]. Activation of IKK upon prolonged TNFα treatment (at least 6 hours) causes phosphorylation of RelB on serine 472 [2]. It allows nuclear ReB to dissociate from its interaction with the inhibitory protein IκBα and to bind to the promoter of pro-migration genes such as MMP3 [3], thereby resulting in selective NF-κB target gene expression involved in the control of TNFα-induced cell migration [4]. TNFα-induced IKK-driven ReB serine-472 phosphorylation is subsequently required for efficient cell migration in an MMP3-dependent manner [5].

Although TNFα is known to induce a massive nuclear accumulation of RelB, it is generally accepted that RelB global DNA-binding activity is not induced upon TNFα treatment in fibroblasts [18]. Our laboratory has recently uncovered that RelB plays a crucial role in promoting fibroblast migration upon prolonged TNFα stimulation. Remarkably, RelB pro-migratory function is driven by its induced phosphorylation on serine 472 [74] (Figure 2). We have identified the two kinases IKKα and IKKβ as novel RelB-interacting partners whose activation by TNFα promotes RelB phosphorylation on serine 472. Moreover, using a custom antiphospho-serine 472-specific RelB monoclonal antibody, we have shown that RelB phosphorylation on serine 472 is induced in fibroblasts in response to both TNFα and PDGFβ [74]. We have demonstrated that nuclear RelB phosphorylated on serine 472 dissociates from its interaction with the inhibitory protein IκBα and binds to the promoter of critical migration-associated genes, such as the metalloproteinase matrix metallopeptidase 3 (MMP3) (Figure 2). Finally, we have shown that RelB serine 472 phosphorylation status controls MMP3 expression and pro-migration activity downstream of TNF receptors (TNFRs) [74] (Figure 2). Interestingly, phosphorylation of RelA on threonine 505, induced by Chk1 kinase, has been reported to inhibit constitutive fibroblast migration [69]. Such observation reinforces the idea of non-redundant functions for RelA and RelB in the control of cell motility. 

### 2.4. Other Putative Phosphorylation Sites

Mass spectrometry approaches have highlighted several other putative sites that can be modified by phosphorylation throughout RelB, such as serine 20, serine 37, serine 116, serine 139, serine 217, tyrosine 293, serine 425, and threonine 579 [77]. Whether phosphorylation of these residues exists *in vivo* and their functional consequences are currently unknown. Nevertheless, it presumes that functional regulation of RelB by phosphorylation is highly complex.

## 3. Polyubiquitination of RelB

In 2008, Leidner *et al.* pinpointed for an ubiquitination-dependent enhancement of RelB transcriptional activity that is not linked to an increase in RelB nuclear localization or DNA binding [73]. Of note, RelB serine 368, serine 552 or threonine 84 (see above) do not seem to be involved in RelB polyubiquitination [73]. RelB ubiquitinylation assays using HA-ubiquitin mutants defective for either Lys^48^ degradative-conjugated polyubiquitin chain or Lys^63^ non-degradative-conjugated polyubiquitin chain, or defective for both, still showed an efficient RelB polyubiquitination. Thus, it indicates that polyubiquitination of RelB might involve other types of polyubiquitin conjugation [78]. Mapping of the ubiquitination target sites revealed the existence of various lysine residues which serve as ubiquitination acceptors throughout the RelB protein. Nonetheless, Lys273/274 and Lys305/308 appeared to be critical for the ubiquitination-dependent increase in RelB transcriptional activity. The nature of polyubiquitin-chain conjugation involved in this process remains unclear. The molecular mechanisms controlling the increase in RelB activity, especially the identity of the recruited co-activators or released co-repressors (e.g., Daxx, EZH2 or G9a) [34,79,80] still need to be explored.

## 4. SUMOylation of RelB

Another post-translational modification reported to modulate the functionality of NF-κB is the conjugation of SUMO peptides at lysine residues, a process that is termed SUMOylation [67]. SUMOylation of a target protein involves the enzymes SUMO-activating protein (E1), the SUMO conjugating protein UBC 9 (E2) and a panel of SUMO ligases (E3), a panel of enzymes quite similar to the ubiquitination machinery. SUMOylation and ubiquitination frequently have antagonistic effects when affecting the function of a particular protein [81].

Seeking a mechanistic explanation for the dual behavior of RelB either as an activator or a repressor of NF-κB target gene expression, Leidner *et al.* have shed light on a SUMOylation-dependent weakening of RelB transcriptional activity. This effect does not rely on changes in RelB nuclear localization or its DNA-binding ability [75]. Mutational analysis of lysine residues throughout RelB revealed that SUMOylation of RelB can occur at numerous sites, and inactivation of seven lysine residues—positions 387, 388, 390, 411, 414, 415, and 416—is required to affect RelB SUMOylation. The mechanism that connects SUMOylation of RelB to a decrease in RelB transcriptional activity is currently unknown. 

## 5. Conclusions

Considering the presence of 22 lysine, 46 serine, 24 threonine and 10 tyrosine residues in human RelB, it is clear that we have just scratched the surface concerning RelB post-translational modification possibilities.

As reviewed here, phosphorylation, ubiquitinylation and SUMOylation have been reported to have an effect on RelB activity, either enhancing or weakening it. Knowing that RelB has been previously shown to behave either as a transcriptional activator or a transcriptional repressor, we can hypothesize that post-translational modifications can be a key determinant to whether RelB will exert an inhibitory or activiation function. Such post-translational-modifications can changing the cofactor that interacts with RelB, leading to a different outcome in the specificity of RelB-dependent nuclear factor κB (NF-κB) response. In the same way, the same cofactor recruited by different post-translational modifications could lead to different target pools of genes, thus conveying on RelB different functions. Furthermore, a modification-dependent RelB degradation could be implicated in determining the duration of the response to a certain stimulus, as its degradation would stop the RelB-dependent response. In support of this hypothesis, Marielfeld *et al.* showed a site-specific phosphorylation on threonine 84 and serine 552 that determines the cleavage and subsequent degradation of RelB [71]. In another study, ReB protein expression levels were shown to control the magnitude of classical NF-κB pathway activation through induced RelB cleavage by the paracaspase MALT1 in B and T cells [76]. However, whether or not in this context RelB post-translational modifications are involved in the control of RelB cleavage and subsequent relief of the classical NF-κB activation pathway is currently unknown. All these possibilities, considered together with all those of other NF-κB family members that interact with and regulate RelB, could explain the versatility of this factor.

We have recently revealed a novel activating molecular mechanism leading to RelB transcriptional activation downstream of TNF receptors. It relies on RelB-serine 472 phosphorylation and is critical for the control of inflammation-induced cell migration [74]. We thus have shed light on a specific RelB post-translational modification that drives RelB to exert a specific biological function. It has been recently reported that RelB can promote the more invasive phenotype of ERα-negative breast cancer cell lines [52], and RelB increases the incidence of metastatic tumors in a mice xenograft model of prostate cancer [51]. Furthermore, RelB knockdown strongly reduces glioma cell migration and invasion [53]. However, whether RelB serine 472 phosphorylation can participate in the invasiveness of cancer cells is currently unknown but is nevertheless worth further investigation.

Unveiling RelB post-translational modifications will provide us not only with a better understanding of the normal regulation of RelB (and the alternative NF-κB pathway), but also with the understanding of its deregulated activity and the pathological consequences that follow. Since this area of research is moving at a rapid pace, there is hope that the processes behind RelB post-translational modifications influencing global NF-κB activity and its involvement in pathological processes will soon be uncovered.

## Figures and Tables

**Table 1:** Post-translational modifications of RelB. The modification, the site(s) involved, the functional effect and reference are indicated in chronological order.

Modification	Site(s)	Enzyme(s)	Effect	Reference
Phosphorylation	Threonine 84, Serine 552	Unknown	Degradation	Marienfeld *et al.* 2001 [71]
Phosphorylation	Serine 368	Unknown	Dimerization	Maier *et al.* 2003 [72]
Polyubiquitination	Lysine 273, 274, 305 and 308	Unknown	Transcriptional activity	Leidner *et al.* 2008 [73]
Phosphorylation	Serine 472	IKKα/IKKβ	Cell migration	Authier *et al.* 2014 [74]
SUMOylation	Lysine 387, 388, 390, 411, 414, 415, and 416	Unknown	Transcriptional activity	Leidner *et al.* 2014 [75]
